# Vivo-Morpholinos Induced Transient Knockdown of Physical Activity Related Proteins

**DOI:** 10.1371/journal.pone.0061472

**Published:** 2013-04-22

**Authors:** David P. Ferguson, Emily E. Schmitt, J. Timothy Lightfoot

**Affiliations:** Department of Health and Kinesiology, Texas A&M University, College Station, Texas, United States of America; University of Nevada School of Medicine, United States of America

## Abstract

Physical activity is associated with disease prevention and overall wellbeing. Additionally there has been evidence that physical activity level is a result of genetic influence. However, there has not been a reliable method to silence candidate genes *in vivo* to determine causal mechanisms of physical activity regulation. Vivo-morpholinos are a potential method to transiently silence specific genes. Thus, the aim of this study was to validate the use of Vivo-morpholinos in a mouse model for voluntary physical activity with several sub-objectives. We observed that Vivo-morpholinos achieved between 60–97% knockdown of Drd1-, Vmat2-, and Glut4-protein in skeletal muscle, the delivery moiety of Vivo-morpholinos (scramble) did not influence physical activity and that a cocktail of multiple Vivo-morpholinos can be given in a single treatment to achieve protein knockdown of two different targeted proteins in skeletal muscle simultaneously. Knocking down Drd1, Vmat2, or Glut4 protein in skeletal muscle did not affect physical activity. Vivo-morpholinos injected intravenously alone did not significantly knockdown Vmat2-protein expression in the brain (p = 0.28). However, the use of a bradykinin analog to increase blood-brain-barrier permeability in conjunction with the Vivo-morpholinos significantly (p = 0.0001) decreased Vmat2-protein in the brain with a corresponding later over-expression of Vmat2 coincident with a significant (p = 0.0016) increase in physical activity. We conclude that Vivo-morpholinos can be a valuable tool in determining causal gene-phenotype relationships in whole animal models.

## Introduction

Physical inactivity has been correlated with cardiovascular disease, obesity, type II diabetes and some types of cancers [1]. With only 3.5% of adults meeting the recommended physical activity guidelines [2], physical inactivity is the second leading actual cause of death (∼250,000 cases/year) in the United States [3] with an estimated $507 billion a year in health care costs [1]. Several potential candidate genes associated with the genetic influence on voluntary physical activity have been identified [1], [4], [5], [6], [7], [8], [9], [10], [11] with potential regulatory effects both in the brain and skeletal muscle.

Of the candidate genes identified, Dopamine Receptor 1 (*Drd1*; [1], [6], [7], [8], [9]), Glucose Transporter 4 [*Glut4*, aka: *Slc2a4*; 11], and Vesicular Monoamine Transporter 2 (*Vmat2*; [4], [10], [12], [13]), have been the most widely studied in association with voluntary physical activity. *Vmat2* is expressed in the brain and skeletal muscle. Studies examining the nucleus accumbens of the brain have shown that *Vmat2* stores dopamine in synaptic vesicles [4], [10], [12], [13]. A loss of *Vmat2* has been associated with a decrease in physical activity and development of Parkinson's disease [10], [13]. Another central factor contributing the regulation of physical activity is *Drd1*; high active mice have an under expression of *Drd1* which results in a decrease in dopamine turnover in the nucleus accumbens [6] and a suggested increase in reward driven behavior and voluntary physical activity [6], [7]. It has recently been proposed that *Drd1* expression in skeletal muscle could have similar effects on physical activity with an increase in D1 receptors (*Drd1* and *Drd5*) in skeletal muscle being associated with an increase in muscle force production and prevention of atrophy [8]. An additional mechanism by which peripheral factors could regulate physical activity is through *Glut4*. Glut4 transports glucose into skeletal muscle [14] and it has been shown that an over expression of *Glut4* is associated with a fourfold increase in voluntary physical activity [11].

Quantitative genetics rarely identify a mechanistic link relating a potential candidate gene to the regulation of a phenotype. To prove causal relationship, transgenic animals with targeted genes knocked-out can be employed. However, a transgenic approach can be confounded by the loss of regulatory regions in the genome as well as developmental issues for the animal [15]. A tool that could transiently silence a specific gene *in vivo* without confounding effects would be ideal in identifying genes that regulate voluntary physical activity and other phenotypes.

Morpholinos are anti-sense oligonucleotide analogs that bind to complementary RNA sequences and inhibit processing of mRNA by blocking translation or splicing of pre-mRNA [16]. While early versions of morpholinos have been in use for over fifteen years with *in vitro* applications, these early morpholino designs presented several problems that prevented use in an *in vivo* model, including rapid degradation by proteases/nucleases [17] and the inability of the morpholino to cross membranes [18]. These problems were solved when Marcos and colleagues [19] altered the delivery moiety of morpholinos by using eight guanidinium head groups of arginine-rich peptides. The resulting oligonucleotide analog, termed “Vivo-morpholinos,” are transported into the cell by endocytosis and protected from proteases and nucleases [19]. Recently there has been evidence suggesting protein knockdown can be achieved equally with intravenous (IV), intraperitoneal (IP), or direct injection into targeted tissue [17], [18], [19], [20].

To date there are only twenty-eight studies using Vivo-morpholinos [17], [18], [19], [20], [21], [22], [23], [24], [25], [26], [27], [28], [29], [30], [31], [32], [33], [34], [35], [36], [37], [38], [39], [40], [41], [42], [43], [44] and all have reported at least 50% knockdown of the target gene with no adverse side effects. Fourteen of these studies used a mouse model [17], [18], [19], [20], [21], [24], [31], [32], [34], [35], [40], [41], [42], [43] with the remaining studies using rats [36], [37], newts [29], chicken embryos [27], fish [15], [16], [22], [23], [25], [26] tadpoles [30], [38], or frogs [28]. In the mouse models, it has been shown that Vivo-morpholinos were equally efficacious with IV or IP, and recent studies have shown success with direct injection in target tissue [36], [37]. While these studies have established the use of Vivo-morpholinos, they were limited in the application parameters used. One of the major limitations of the initial validation studies was the short-term nature of the studies (e.g. 24–48 hours post treatment) even though it has been suggested [19] that gene silencing with Vivo-morpholinos will theoretically last much longer (e.g. 7 days). Further, the existing Vivo-morpholino studies have only evaluated effects of a single Vivo-morpholino without indication of whether multiple genes can be silenced simultaneously. Additionally, there has been little success in applying Vivo-morpholinos to the mouse brain [19].

Therefore, the purpose of this study was to validate the use of Vivo-morpholinos in silencing targeted genes in a mouse model of voluntary physical activity. This study included evaluation of the appropriate control, the effectiveness of transporting a Vivo-morpholino across the blood brain barrier (BBB) with a pharmacological aid, the ability to combine multiple Vivo-morpholinos in a “cocktail” to silence multiple genes simultaneously, and whether daily physical activity altered the washout time course of an intravenous injected Vivo-morpholino.

## Methods

Three separate experiments were used to fulfill the purposes of this project. All experiments were approved by the Texas A&M Institutional Animal Care and Use Committee (Animal use protocols 2010-256, 2010-187, 2011-140, and 2011-147) and all animals were housed in an AAALAC certified vivarium on a 12-hour light/dark cycle with *ad libitum* access to standard chow and water. Animals were monitored and efforts were taken to ameliorate any animal suffering. All experiments used Vivo-morpholinos ordered in the 400-nmole batches.

### • Experiment 1: Evaluation of Vivo-morpholinos' gene silencing in mouse activity model

This experiment evaluated the effectiveness of silencing genes in a physical activity model using Vivo-morpholinos, a method of Vivo-morpholino delivery across the BBB, as well as the appropriate control vehicle. Eighteen male C57Bl/6J mice (Jackson Labs, Bar Harbor, ME) were randomly assigned to one of three treatment groups:

Group 1) intravenously injected a translation-blocking Vivo-morpholino (11 mg/kg) [19]; Gene Tools LLC, Philomath, OR) targeting *Vmat2* (**Vmat2** group, n = 6);

Group 2) Intravenously injected a Vivo-morpholino scramble control (11 mg/kg) which consisted of vehicle plus an oligonucleotide target (5′-CCTCTTACCTCAGTTACAATTTATA-3′) that did not correspond to murine mRNA (**Scramble** group, n = 6); and

Group 3) An intravenously injected control group that received an equal volume (110 ul) of physiological saline (**Saline** group, n = 6).

Preliminary experiments determined that systemically delivered Vivo-morpholinos did not knockdown brain proteins [45]. Thus, to facilitate Vivo-morpholino transport across the BBB, all treatment groups received the bradykinin analog RMP-7 (6.5 µg/kg; Bachem, Prussia, PA) [46]. Pharmacological studies have shown that using endogenous analogs of bradykinin [47] increase permeability of the BBB while maintaining physiological function of the animal [46]. Bradykinin activates β2 receptors on endothelial cells of cerebral capillaries thereby disengaging tight junctions and causing an increase in permeability of cerebral blood vessels [46], [47]. RMP-7 is a bradykinin analog that has several benefits over bradykinin; specifically RMP-7 resists degradation, has little to no toxicity to the brain, can be administered intravenously, and is fast acting producing a result within 60 seconds of administration [47]. RMP-7 was initially developed as a delivery method for chemotherapeutics in brain glioma patients with studies showing a linear dose response between RMP-7 and BBB permeability in conjunction with a variety of chemotherapeutics [46].

At eight weeks of age mice were individually housed with running wheels equipped with computers (Sigma Sport, St. Charles, IL) to measure average daily distance run [48], and beginning at nine weeks of age, completed a week of baseline wheel running. At ten weeks of age, mice were randomly assigned to one of the three treatment groups and received a tail vein injection of the specific treatment for three consecutive days. At 11 weeks of age (4 days post the last injection) half the cohort was sacrificed (n = 3 per treatment group). The activity of the remaining mice was monitored for an additional week (recovery) and the mice were then sacrificed.

At sacrifice, mice were anesthetized using vaporized isoflurane followed by cervical dislocation. The soleus (peripheral slow twitch muscle) and nucleus accumbens (reward center of the brain) were removed and flash-frozen for later analysis. Gene silencing was evaluated by determining protein knockdown using standard SDS-Page and Western blotting techniques. Briefly, proteins were extracted by placing the tissue in lysis buffer and homogenizing the tissue with a motor and pestle. Protein concentration was determined by Bradford assay to ensure equal amount of protein loading per sample on the gel. The proteins were separated by SDS-Page, and then transferred to a nitrocellulose membrane with transfer confirmed by *Ponceau S* stain. Membranes were incubated overnight in 1∶1000 ratio of primary antibody recognizing Vmat2 (Cell Applications, San Diego, CA) and blocking buffer (5% Nonfat Dried Milk, 0.5% Tween 20). Membranes were then incubated in the secondary horseradish peroxidase antibody (Cell Signaling Technology, Beverly, MA). Chemiluninescence was imaged with a Flurochem analyzer (Derbyshire, UK) and the blot was analyzed using the individual protein band's optical density that allowed for a semi-quantitative estimate of protein knockdown.

The Western blot optical densities were analyzed using one-way ANOVA to compare Vmat2 protein expression between the treatments with an *a priori* alpha level of 0.05. Activity data was analyzed using a 3×3 ANOVA with the main effects being treatment group (i.e. **Vmat2**, **Saline**, or **Scramble**) and week of treatment (i.e. baseline, injection, or recovery). If there was a significant main-effect, a Tukey's HSD *post hoc* (p<0.05) test was employed. All statistical tests were carried out using GraphPad Prism 5 (GraphPad Software Inc, La Jolla, CA).

### • Experiment 2: Determination of Vivo-morpholino Washout Period

To evaluate if physical activity altered the efficacy of the Vivo-morpholino, 24 eight week old C57Bl/6J male mice (Jackson Labs, Bar Harbor, ME) were individually housed and randomly assigned to either a running wheel group (n = 12; unlocked wheels) or a fixed wheel group (n = 12; locked wheels). The running wheel group had free moving wheels attached to a computer as described in Experiment 1. Each mouse in the fixed running wheel group had a running wheel that was secured to prevent wheel movement. Beginning at nine weeks of age, daily distance was measured in the running wheel group using methods from Experiment 1. At ten weeks of age one mouse from each group was sacrificed to establish a baseline level of Vmat2 protein expression, while the remaining mice received a concurrent tail vein injection of RMP-7 (6.5 ug/Kg) [46] and a Vivo-morpholino targeting *Vmat2* (11 mg/Kg) [19] for three consecutive days. Each day thereafter, one mouse was sacrificed from each group to determine the washout of the Vivo-morpholino and whether exposure to activity affected protein knockdown.

On the day of sacrifice, mice were euthanized using vaporized isoflurane and cervical dislocation, with subsequent harvesting of the soleus and nucleus accumbens. Afterwards, proteins were extracted from each sample and Vmat2 expression, as well as α-synuclein (Cell Signaling, Danvers, MA), which has been shown to be an indicator of *Vmat2* transcription [49] were determined using Western Blot techniques described in Experiment 1. The washout data was then analyzed for linearity and if nonlinear, nonlinear regression approaches were used to obtain R^2^ values for the washout curves. The nonlinear data was then log transformed to meet the parameters of a linear regression, which allowed the comparison of the regression parameters between the locked and unlocked running wheel groups. A p value of 0.05 was set *a priori* to determine if the slopes and y intercepts were different from each other. If there were no differences between the locked and unlocked wheel groups, the data points were pooled. To facilitate the comparison of baseline physical activity across the treatment protocol, average daily distance measurements were pooled in animals sacrificed in days 1–5 and days 6–10 of the protocol and compared using a one-way ANOVA (*a priori* alpha value = 0.05).

### • Experiment 3: Utilization of a Vivo-morpholino Cocktail

Twelve, eight-week old male C57Bl/6J mice (Jackson Labs, Bar Harbor, ME) were individually housed with running wheels as described in Experiment 1 to measure average daily distance run. At nine weeks of age mice completed a week of baseline wheel running and then at ten weeks of age, randomly received a tail vein injection of either the Vivo-morpholino cocktail (n = 6) containing a Vivo-morpholino targeting *Drd1* (11 mg/kg) [19] and another Vivo-morpholino targeting *Glut4* (11 mg/kg) [19] or physiological saline (n = 6) for three consecutive days. Physiological saline was used as a control since Experiment 1 showed no difference in the activity response of physiological saline and Vivo-morpholino scrambled control (see Results). At 11 weeks of age (four days post-last injection), half the cohort was sacrificed to evaluate the initial protein knockdown efficacy of the Vivo-morpholino cocktail. The remaining mice ran for one week and were then sacrificed. Upon sacrifice, the soleus was harvested and protein expression of Drd1 (Cell Applications, San Diego, CA) and Glut4 (Cell Applications, San Diego, CA) as well as the potential compensatory proteins dopamine receptor 5 (Drd5, Proteintech Group Inc, Chicago, IL) and glucose transporter 1 (Glut1, Cell Applications, San Diego, CA) were determined using standard Western blotting techniques, as described in Experiment 1. Similar statistical analysis was used as in Experiment 1 with the exception of a 2×3 ANOVA with the main effects being treatment (Saline or Vivo-morpholino cocktail) and treatment week (Baseline, Injection, or Recovery).

## Results

### • Experiment 1: Evaluation of Vivo-morpholinos in activity model

**Figure 1:**
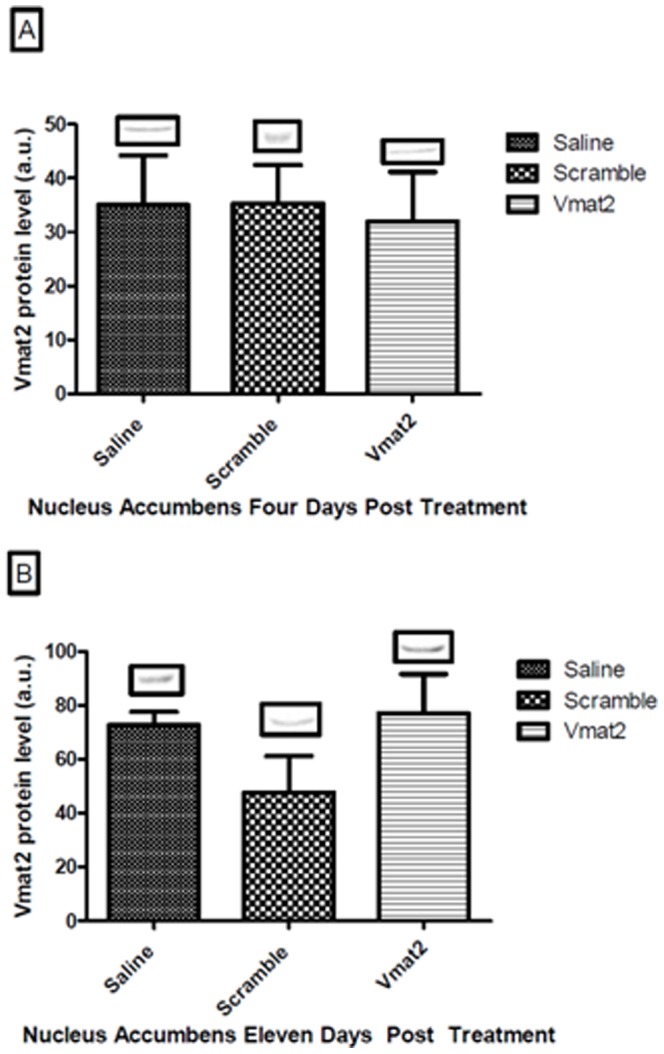
Experiment 1, Vmat2 protein level in the nucleus accumbens. Mean ± standard deviation of western blot optical band density and a representative western blot comparing Saline, Scramble and Vivo-morpholino treated animals for Vmat2 protein expression in the nucleus accumbens of the brain. There was no difference between the treatment groups at four (p = 0.87, Panel A) or eleven (p = 0.14, Panel B) days post treatment.

**Figure 2:**
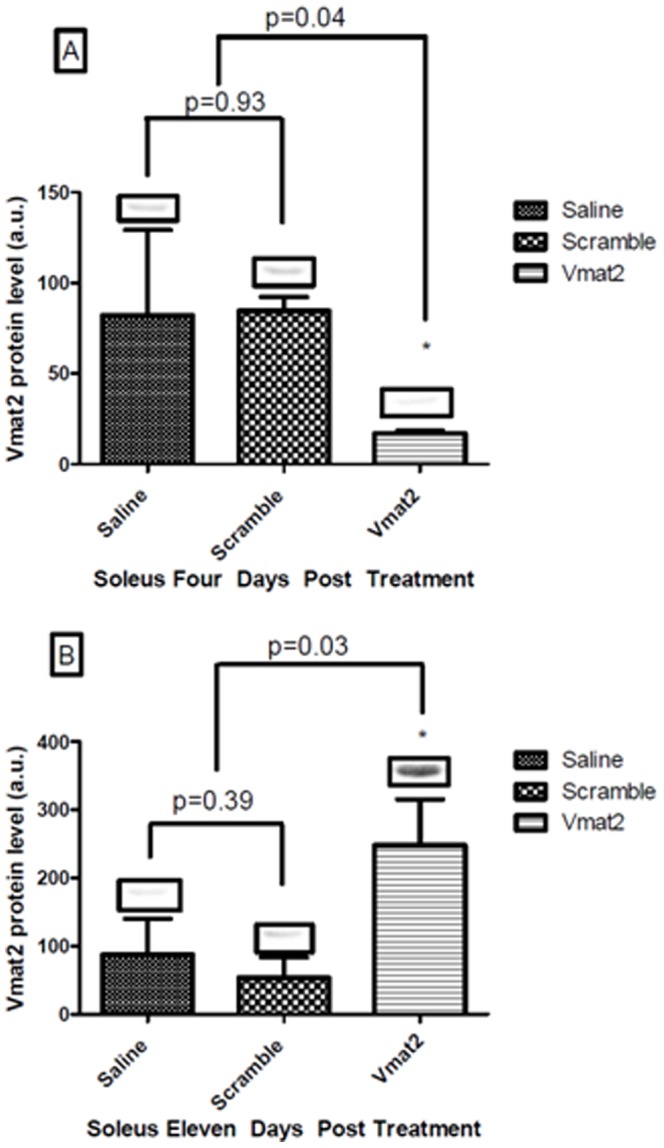
Experiment 1, Vmat2 protein level in the soleus. Mean ± standard deviation of western blot optical band density and a representative western blot comparing Saline, Scramble and Vivo-morpholino treated animals for Vmat2 protein expression in the soleus. Panel A shows Vmat2 expression at four days post-last injection. There was a significant decrease (p = 0.04) in Vivo-morpholino treated animals compared to Saline and Scramble treated animals in Vmat2 expression at four days post injection. Panel B shows Vmat2 expression at eleven days post-last injection. There was a significant increase (p = 0.03) in Vivo-morpholino treated animals compared to Saline and Scramble treated animals in Vmat2 expression at eleven days post injection.

In the brain, treatment with a Vivo-morpholino targeting *Vmat2* did not result in a significant knockdown of Vmat2 expression in the nucleus accumbens of the brain four days after the last morpholino injection as compared to saline or scramble treatment (Figure 1, Panel A). However, there was a 79% knockdown (p = 0.04) of Vmat2 in the soleus four days post-injection as compared to both saline and scramble (Figure 2, Panel A). Surprisingly, during the recovery week (11 days post treatment) Vmat2 protein level had a significant 354% over expression in the soleus of the **Vmat2**-Vivo-morpholino group compared to the saline and scramble treatment (Figure 2, Panel B, p = 0.03). In the nucleus accumbens (Figure 1) and the soleus (Figure 2) there was no difference in Vmat2 expression between saline and scramble treatments.

**Figure 3:**
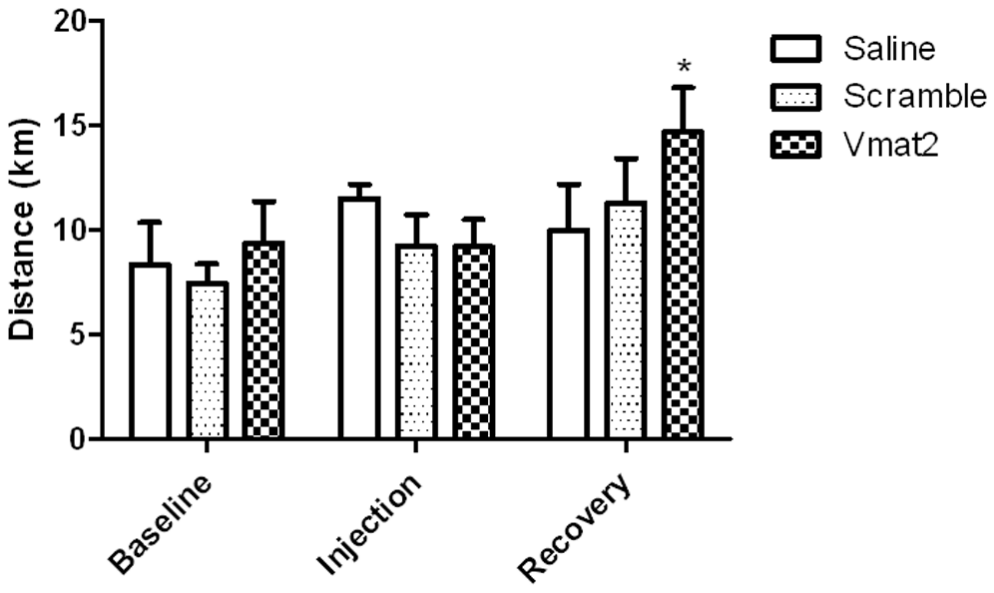
Experiment 1, mouse wheel running. Average daily distance ran for the baseline, injection, and recovery week for animals treated with saline, Vivo-morpholino vehicle only (scramble) and the Vivo-morpholino targeting *Vmat2* (Vmat2). Vmat2 group significantly (*p = 0.001) increased activity in the recovery week compared to the baseline and injection week. There was no difference in physical activity (p>0.05) in the scramble or saline treated animals for baseline, injection, and recovery weeks.

The initial reduction of Vmat2 in the soleus did not affect daily physical activity (Figure 3). However, the **Vmat2** group had a significant 139% increase in activity during the recovery week as compared to the baseline and injection week (Figure 3, p = 0.001), which corresponded to the Vmat2 over-expression observed in the soleus eleven days post-injection (Figure 2, Panel B).

### • Experiment 2: Determination of Vivo-morpholino Washout Period

**Figure 4:**
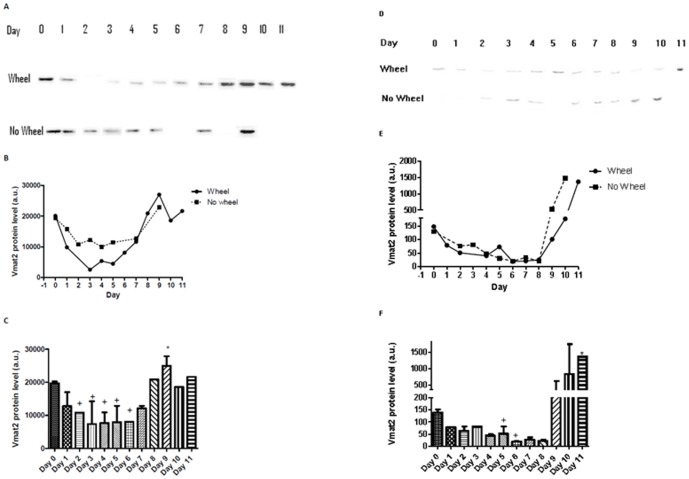
Experiment 2, Evaluation of the washout time course of Vivo-morpholinos targeting *Vmat2*. Panels A, B, and C represent soleus samples while panels D, E, and F represent nucleus accumbens samples. Panel A represents western blot data probing for Vmat2 in the soleus for both unlocked wheel and locked wheel groups. Panel B is the optical density of the individual western blot bands from panel A. A nonlinear regression was run on the data in panel B which generated an R^2^ = 0.65 for the animals on unlocked wheels and an R^2^ = 0.55 for animals on locked wheels. Given there was no difference (p = 0.42) in the slope or y intercept of the unlocked wheel and locked wheel groups, the data were pooled at each Day (Panel C). Panel C shows that there was a significant knockdown (^+^p = 0.001) in Vmat2 on days 2–6 and a significant (^*^p = 0.001) over expression in Vmat2 on day 9 in the soleus. The same methodologies were applied to Panels D, E, and F for the nucleus accumbens. The nonlinear regression resulted in R^2^ = 0.99 for the unlocked wheel group and R^2^ = 0.99 for the locked wheel group, and there was no difference (p = 0.66) in the slope and y intercepts of the lines in panel E. Additionally Vmat2 was only significantly knocked down on Days 5 and 6 (^+^p = 0.001) with a significant over expression (^*^p = 0.001) on Day 11. The mouse in the locked wheel group representing data for Day 11 was removed from analysis because it developed malocclusion in the front incisors and became malnourished, thus altering physical activity level.

The evaluation of the washout time course of Vivo-morpholinos targeting *Vmat2* showed there was a significant (p = 0.001) 55% knockdown of Vmat2 in the soleus on Days 2–6 (Figure 4 Panels A and C) with a significant 129% over expression observed on Day 9 (p = 0.001). There was no difference (p = 0.74) between the washout curves for the animals who had access to a running wheel (R^2^ = 0.65) compared to those that had a locked running wheel (R^2^ = 0.55) (Figure 4 Panel B).

In the nucleus accumbens, there were similar observations as in the soleus. There was a significant 74% knockdown in Vmat2 (Figure 4 Panels D and F) on Days 5–6 (p = 0.001) with a 988% over expression on Day 11 (p = 0.001). Additionally there was no difference in the washout curves (p = 0.66) for animals on wheels (R^2^ = 0.99) or locked wheels (R^2^ = 0.99) (Figure 4 Panel E).

**Figure 5:**
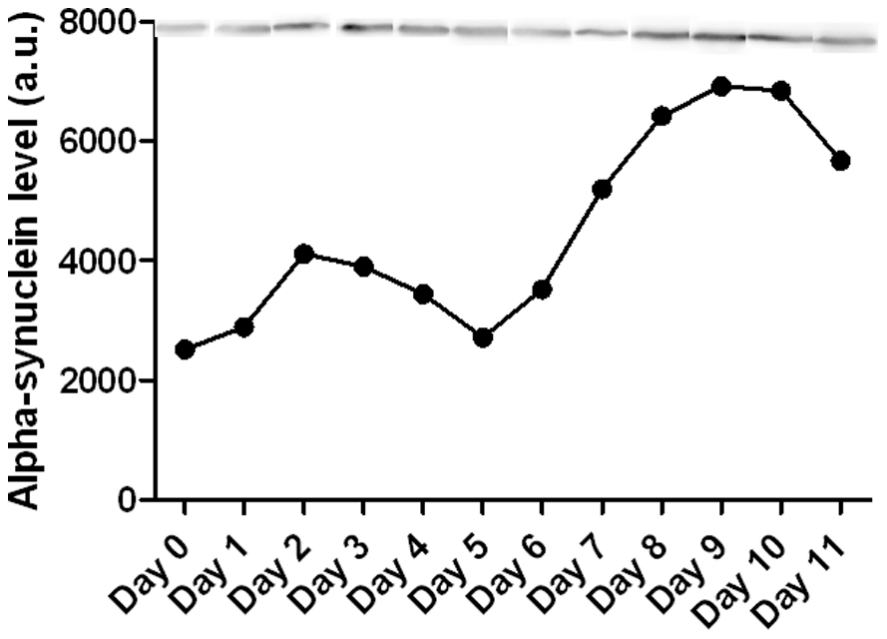
Experiment 2, α-synuclein protein level in nucleus accumbens. Western blot analysis probing for α-synuclein to confirm the signal for the over expression of Vmat2 protein seen in the nucleus accumbens. There was an increase in α-synuclein at days 2 and 7–10. α-synuclein is an indicator of *Vmat2* transcription thus when Vmat2 was knocked down by the Vivo-morpholino there was an increased stimulus placed on the cell to transcribe more *Vmat2*.

To confirm the over expression of Vmat2, western blot analysis was performed probing for α-synuclein, which is an indicator of *Vmat2* transcription [49]. The results showed that in the brain on Days 2 and 7 there was a 275% increase α-synuclein protein level (Figure 5).

**Figure 6:**
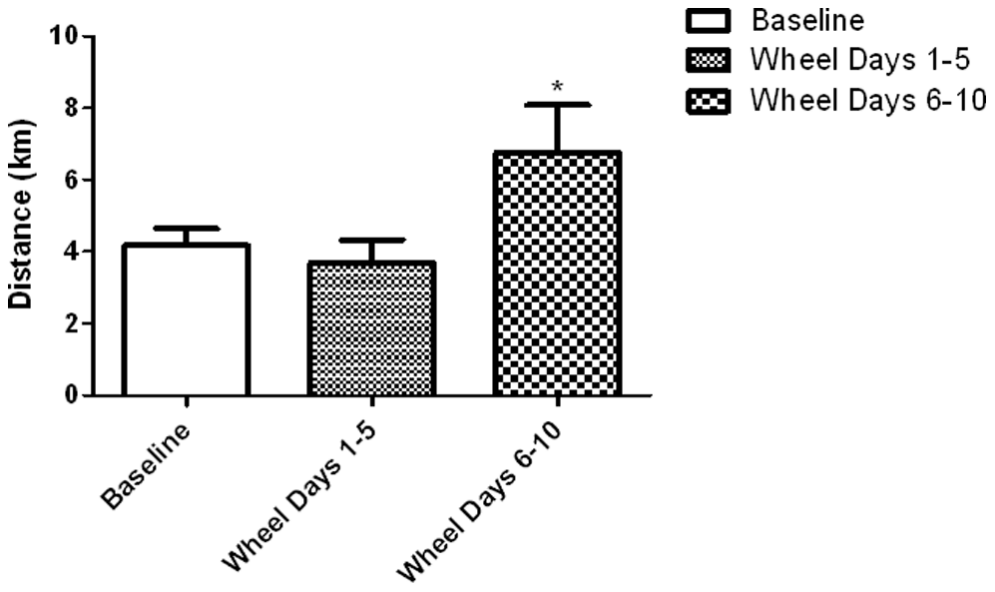
Experiment 2, mouse wheel running. Average daily distance ran for animals treated with Vivo-morpholino targeting *Vmat2* for the baseline week, injection week (Days 1–5), and recovery week (Days 6–10) of washout protocol. Physical activity was significantly (*p = 0.0016) increased in Days 6–10.

There was no difference (p>0.05) in physical activity level of the running wheel group between the baseline week and days 1–5 post-injection (Figure 6). During days 6–10 of the washout protocol there was a significant 164% increase in physical activity (p = 0.0016) versus baseline and days 1–5, corresponding to the increase in Vmat2 expression during the same time period (Figure 4).

### • Experiment 3: Utilization of a Vivo-morpholino Cocktail

**Figure 7:**
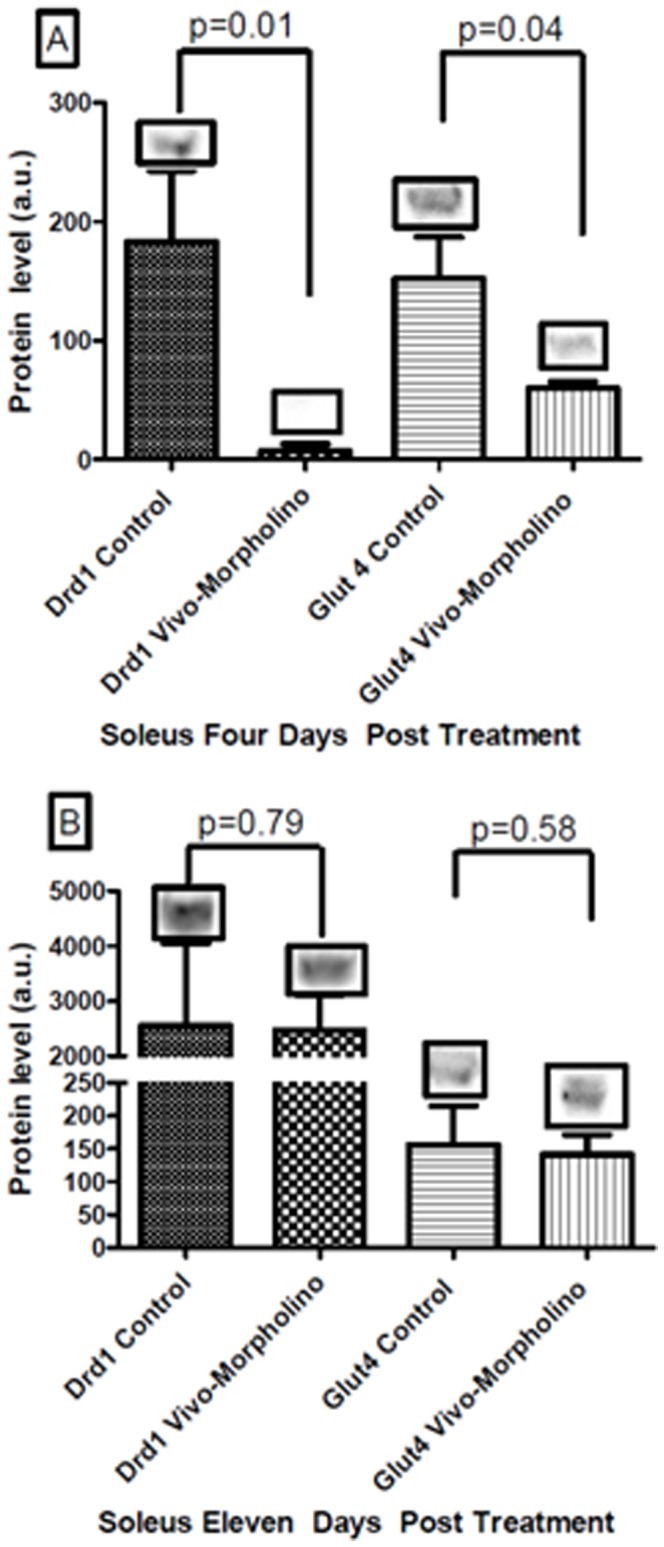
Experiment 3, Drd1 and Glut4 protein level. Western blot and mean ± standard deviation of optical densities of soleus tissue that underwent the Vivo-morpholino cocktail treatment. Panel A represents four days post treatment of Drd1 and Glut4 expression comparing controls to Vivo-morpholino cocktail treated animals. There was a significant knockdown for both Drd1 (p = 0.01) and Glut4 (p = 0.04). Panel B represents eleven days post treatment of Vivo-morpholino cocktail. There was no difference between control and Drd1 (p = 0.79) and Glut4 (p = 0.58) expression.

**Figure 8:**
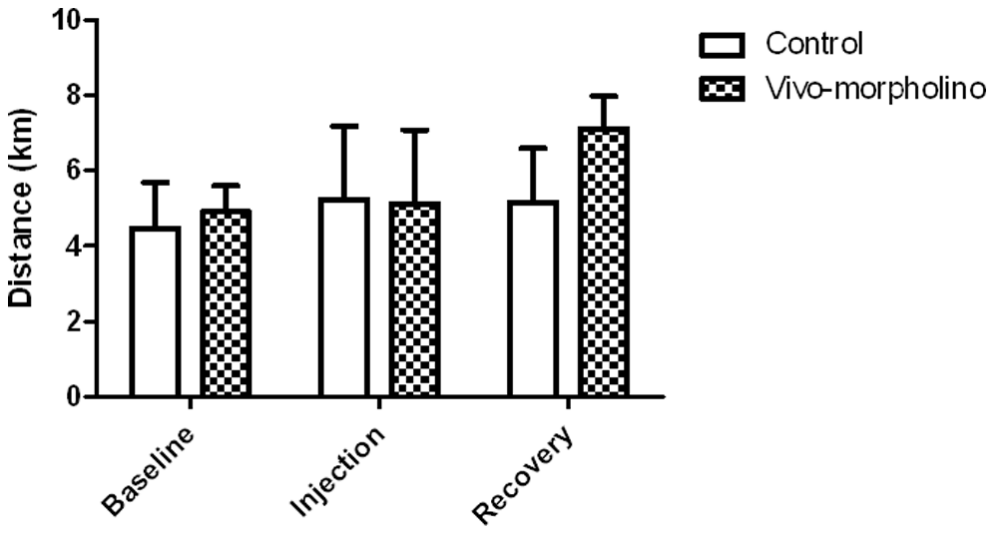
Experiment 3, mouse wheel running. Average daily distance ran for baseline, injection, and recovery week for animals treated saline (control) and the morpholino cocktail targeting Drd1 and Glut4 (Vivo-morpholino). There was no difference (p = 0.15) between the activity of the control group and the morpholino group.

Western blot analysis showed that there was significant knockdown in Drd1 (97%) protein (Figure 7, Panel A, p = 0.01) and Glut4 (60%) protein (Figure 7, Panel A, p = 0.042) in the soleus four days after the last injection. Expression of both Drd1 and Glut4 returned to control levels during the recovery week (11 days post injection, Figure 7, Panel B). With the Vivo-morpholino cocktail treatment (*Drd1* and *Glut4*) there was no change in physical activity at any time period (Figure 8, p = 0.15).

**Figure 9:**
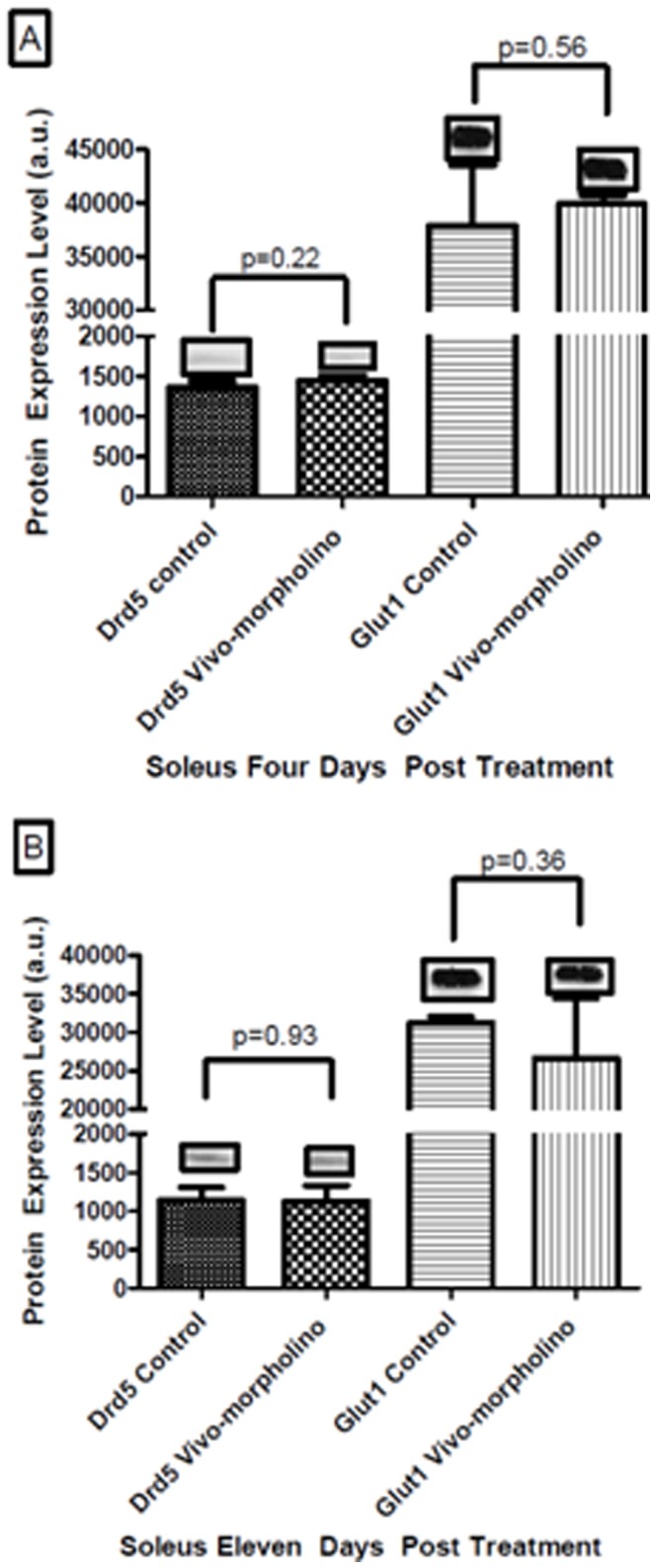
Experiment 3, Drd5 and Glut1 protein level. Western blot and mean ± standard deviation of optical densities of soleus tissue that underwent the Vivo-morpholino cocktail treatment. Panel A represents four days post treatment of Drd5 and Glut1 protein expression comparing controls to Vivo-morpholino cocktail treated animals. There was not a significant knockdown for either Drd5 (p = 0.22) or Glut1 (p = 0.56). Panel B represents eleven days post treatment of Vivo-morpholino cocktail. There was no difference between control and Drd5 (p = 0.93) and Glut1 (p = 0.36) expression.

To evaluate potential compensation for the decreases in Drd1 and Glut4, Drd5 and Glut1 were probed by western blotting (Figure 9). There was no difference in Drd5 protein levels between control and treatment at four days post treatment (p = 0.22) or eleven days post treatment (p = 0.93). There was also no difference in Glut1 protein levels between control and treatment at four days post treatment (p = 0.56) or eleven days post treatment (p = 0.36).

## Discussion

A molecular biology tool that can transiently silence genes in a whole-animal model will significantly advance the capability to determine cause-effect relationships between specific genes and targeted phenotypes. We have found that the use of Vivo-morpholinos targeting *Vmat2*, *Drd1*, and *Glut4* resulted in transient protein knock-down in the skeletal muscle up to four days after the last application. While the use of a pharmacological agent to facilitate Vivo-morpholino delivery across the blood brain barrier seemed promising, our results indicate marginal knockdown in the brain as compared to the soleus when using RMP-7. Further, we observed that concurrent physical activity did not influence the action of Vivo-morpholinos on Vmat2 protein expression. Maybe most importantly, we also observed that the use of multiple Vivo-morpholinos in a cocktail would knock down more than one protein at a time in skeletal muscle. Interestingly, while initial knockdown of these proteins did not affect physical activity, we observed an increased physical activity coincident with post-washout over-expression of Vmat2.

The search for an *in vivo* technique to transiently silence genes has been pursued for several decades with numerous promising tools identified including antisense oligonucleotides, small interfering RNA (siRNA), and phosphorodiamidate morpholino oligomers (PMO). Antisense oligonucleotides are effective at gene silencing yet are highly unstable in body fluids and are rapidly degraded [17]. Initially, siRNA promised to transiently silence genes but presented difficulty with *in vivo* applications [44], which has limited siRNA's usefulness in integrative physiology. PMOs resist degradation *in vivo* yet have been associated with increased toxicity [17]. Morpholinos, which are a form of antisense oligonucleotides, have had several modifications to their structure to limit toxicity and improve gene silencing which led to the development of the more stable Vivo-morpholino [19]. Additionally, the fact that we observed no change in daily physical activity with the vivo-morpholino delivery vehicle (scramble) makes the Vivo-morpholino ideal for the investigation of candidate genes associated with physical activity.

Our results showed that *Drd1-*, *Glut4-*, and *Vmat2*-targeted Vivo-morpholinos significantly knocked down Drd1, Glut4, and Vmat2 protein expression in skeletal muscle by an average 97%, 60%, and 79%, respectively. Furthermore, the use of the Vivo-morpholino cocktail showed that simultaneous protein knockdown was possible. Previous studies have shown that treatment with Vivo-morpholinos at a similar dosage as used in this study (i.e. 11 mg/kg) resulted in 50–70% knockdown [16], [26], similar to the knockdown amounts we observed. Recent literature [43] suggests that to obtain 100% protein knockdown, the Vivo-morpholino dosage must be 160% greater than the amount prescribed by the manufacturer (i.e. 17.6 mg/kg) or the Vivo-morpholino injections must be given regularly over the course of the study [17], [20]. The fact that there were differences in the magnitude of Drd1 and Glut4 knockdown suggests that the nature of the target gene also may play a role in the magnitude of gene silencing. Genes coding for proteins that exhibit faster turnover rates in skeletal muscle may require the higher Vivo-morpholino dose. Therefore, protein turnover characteristics should be considered as a design parameter in Vivo-morpholino protocols.

Our initial results with Vivo-morpholinos in brain tissue were not promising due to a potential lack of BBB penetration [45]. Vivo-morpholinos are ∼10,000 Daltons in size and the BBB prevents substances greater than 400 Daltons from reaching the brain [46], thus we utilized RMP-7 as means to facilitate Vivo-morpholino delivery across the BBB. Unfortunately, in spite of the potential benefits of using RMP-7, Experiment 1 did not show significant knockdown in the nucleus accumbens of Vmat2 protein when using RMP-7 on day four after the injection. Interestingly, we did observe Vmat2 knockdown in the nucleus accumbens in Experiment 2 on Days 5 and 6 after the last injection, which was outside of our measurement range in Experiment 1. We suspect that due to the high activity of the dopamine system in the brain [50], [51], [52], there may be a decreased protein turnover rate of Vmat2 in the brain as compared to skeletal muscle [50], [53], [54], [55] resulting in a slower protein turnover rate leading to a slower knockdown time course of Vmat2 in the brain. Thus, while the use of RMP-7 appears to have facilitated some knockdown of Vmat2 in the brain, knockdown of brain genes using vivo-morpholinos needs to be carefully designed to optimize both knock-down magnitude and time-course.

Surprisingly, in both Experiments 1 and 2, we observed a rebound over expression of Vmat2 protein compared to baseline during the recovery week in both skeletal muscle and nucleus accumbens. To our knowledge, this rebound phenomena has not been reported in the Vivo-morpholino literature. We would suggest that this rebound over-expression was actually the result of the feedback loop that controls Vmat2 [55]. Speculatively, with the blocking of *Vmat2* mRNA and a reduction of Vmat2 protein concentration, the cell would have been stimulated to transcribe more *Vmat2*. With subsequent Vmat2 production being blocked by the Vivo-morpholino, the stimulus to produce more *Vmat2* mRNA would become further elevated compared to the initial days of treatment. Once the Vivo-morpholino was degraded and removed, the elevated signal to produce Vmat2 would result in an increased translation of *Vmat2* mRNA [55] and over-expression of Vmat2 protein. This hypothesis is supported by our observation of a significant increase in α-synuclein in the nucleus accumbens on Day 2, with a continued increased signaling from Day 7 and peaking on Day 10 (Figure 5). Whereas α-synuclein has been observed to be an indicator of the cell's stimulus to transcribe *Vmat2* [49], it appears that once Vmat2 was knocked down there was an increase in the stimulus to transcribe *Vmat2* which resulted in the over expression of Vmat2 in the recovery week. While we did not observe a rebound over expression with either of the other genes we investigated (Drd1 and Glut4), the possibility of such a rebound effect occurring with other genes is potentially an intriguing way to use Vivo-morpholinos to explore consequences of both gene under expression and over expression in the same model.

It has been previously suggested that *Drd1*, *Glut4*, and *Vmat2* are potential candidate genes for the regulation of physical activity [1], [6], [7], [8], [10], [13] with *Glut4* in particular, showing a direct effect on activity when over expressed [11]. Thus, we were somewhat surprised that knockdown of both Drd1 and Glut4 simultaneously in soleus (Experiment 3) did not affect activity levels. However, our observed lack of effect of Glut4 and Drd1 knockdown on physical activity in spite of an observed 60% and 97% protein reduction, respectively, could have reflected a general redundancy in these physiological systems. For example, it has been shown that with a 50% reduction in *Glut4* there is no apparent change in muscle physiology because of a compensatory *Glut1* over-expression for the loss of *Glut4* [56], [57]. Therefore, we evaluated possible compensation in these two systems by analyzing Drd5 and Glut1 protein levels. We observed no differences in Drd5 or Glut1 levels between control and treatment groups suggesting that compensation for the knock down of Glut4 or Drd1 did not occur and therefore, neither Glut4 nor Drd1 are primary peripheral regulating genes in voluntary activity. However, in spite of these results, it is clear that future studies can take advantage of the use of vivo-morpholino cocktails to silence multiple genes at once to consider their phenotypic effects.

Vmat2 was an attractive knockdown target for the regulation of physical activity given its proven role in the development of Parkinson disease [10]. However, we observed no change in activity with a decrease in peripheral Vmat2 in Experiments 1 and 2. However, the rebound of Vmat2 observed in the soleus (Experiment 1 and 2) and nucleus accumbens (Experiment 2) along with the associated increase in physical activity could provide a potential explanation for the regulation of voluntary physical activity. It has been shown that the dopamine system in skeletal muscle affects muscle force production [8] and in the brain affects reward driven behavior [6]. When mice are given, artificial dopamine there is an associated hyperactivity response [50], [51], [54], [58]. We would suggest that as the effects of the Vivo-morpholinos wore off and *Vmat2* expression returned and surpassed baseline, there was an increase in extracellular dopamine that elicited the hyperactive effects similar to artificially administering dopamine. Further, this extracellular dopamine would have increased skeletal muscle tone and force production [8], [59] as well as reward driven behavior [6], which would have further increased physical activity.

The purpose of these experiments was to evaluate the efficacy of a potential transient gene-silencing tool, the Vivo-morpholino, when used in a physical activity model for an extended period of time. We observed that Vivo-morpholinos that targeted *Vmat2*, *Drd1* or *Glut4* significantly reduced associated protein expression in skeletal muscle up to four days after treatment with a subsequent recovery to baseline levels in Drd1 and Glut4 and with an over expression in Vmat2. Physical activity during the treatment did not affect the time course of protein knockdown. The delivery vehicle used (scramble Vivo-morpholino) did not affect protein knockdown or physical activity. Further, we found that multiple Vivo-morpholinos given in a cocktail knocked down multiple proteins simultaneously. While knocking-down any of the targeted genes did not result in hypothesized reductions in physical activity, a rebound of Vmat2 protein was associated with an increase in physical activity during the same time as the rebound. We conclude that the use of Vivo-morpholinos can be a powerful tool to transiently silence specific genes and determine whether those genes are causally related to a phenotype of interest, especially when the targeted gene's protein characteristics, such as turnover rate and location, are used to strengthen the research design.
